# Editorial: Engineering Yeast to Produce Plant Natural Products

**DOI:** 10.3389/fbioe.2021.798097

**Published:** 2021-12-02

**Authors:** Yongjun Wei, Boyang Ji, Rodrigo Ledesma-Amaro, Tao Chen, Xiao-Jun Ji

**Affiliations:** ^1^ Key Laboratory of Advanced Drug Preparation Technologies, Ministry of Education, School of Pharmaceutical Sciences, Zhengzhou University, Zhengzhou, China; ^2^ Laboratory of Synthetic Biology, Zhengzhou University, Zhengzhou, China; ^3^ Department of Biology and Biological Engineering, Chalmers University of Technology, Gothenburg, Sweden; ^4^ Department of Bioengineering and Imperial College Centre for Synthetic Biology, Imperial College London, London, United Kingdom; ^5^ Frontier Science Center for Synthetic Biology and Key Laboratory of Systems Bioengineering (Ministry of Education), Tianjin, China; ^6^ SynBio Research Platform, Collaborative Innovation Center of Chemical Science and Engineering, School of Chemical Engineering and Technology, Tianjin University, Tianjin, China; ^7^ College of Biotechnology and Pharmaceutical Engineering, Nanjing Tech University, Nanjing, China

**Keywords:** synthetic biology, yeasts, plant natural products, metabolic engineering, *Saccharomyces cerevisiae*

Plants produce diverse natural products, and some of them are drugs or drug precursors. Especially, traditional Chinese medical herbs and other medical plants have the capacity to produce a wide range of bioactive compounds. The extraction of these natural products from plants requires substantial time, land and water usage, and they often produce with low yields. In addition, production can be variable, being affected by pests or climate changes. These factors result in the limited supply of plant bioactive compounds at affordable costs. Therefore, it is necessary to develop efficient and eco-friendly alternative production strategies.

Many yeasts can be genetically manipulated, and thanks to efficient tools and strategies of molecular biology, their metabolism can be reprogrammed (Nielsen and Keasling, 2016; Ji et al., 2020). Achievements of microbial engineering, such as the production of artemisinin (Paddon et al., 2013) and ginsenosides (Wang et al., 2015), have suggested that yeasts can be ideal microbial cell factories for the synthesis of plant natural products. However, understanding the plant biosynthetic pathways and engineering yeasts for production present some bottlenecks and challenges, such as the current limitations on enzyme discovery tools and high-throughput engineering strategies.

In this research topic, the tools and strategies for yeast engineering, and their applications for the production of several plant natural products were described. The discovery and application of CRISPR/Cas systems in microorganisms is revolutionizing the strain design (Pickar-Oliver and Gersbach, 2019). In this topic, Shan et al. summarized the different optimized strategies for CRISPR/Cas systems and their applications in the construction of non-conventional yeast-based cell factories. Zhang and Shi reviewed the recent applications of transcription factor (TF) based biosensors to dynamically control the production of natural products in yeasts. The biosensors targeting to intermediates in natural product synthesis pathways (i.e., fatty acid synthesis, shikimate pathway, and mevalonate pathway) can be further implemented for improving the biosynthetic efficiency of plant natural products. Recent studies have shown that metabolic mass transfer is one of important factors to improve the heterologous production in microorganisms (Ma et al., 2021a). In relation to this, Xue et al. summarized the diverse strategies used for metabolic mass transfer during the production of plant natural products using engineered yeasts, and covered how properly refining/balancing metabolic flux with the metabolic mass transfer strategies would further enhance the biosynthesis efficiency. Tan et al. introduced their approach using efficient selection scheme for the incorporation of non-canonical amino acids into *Saccharomyces cerevisiae* proteins. This efficient selection scheme will expand the application of non-canonical amino acids for protein engineering in yeast cell factories.

Plant terpenoids are one of the main sources of bioactive compounds with pharmaceutical applications. Several bioactive terpenes have been produced using engineered *S. cerevisiae* and other yeasts (Yang et al., 2020; Ma et al., 2021b). Guan et al. described the discovery of glycyrrhetinic acid biosynthetic pathway and summarized the development of its biosynthesis using engineered *S. cerevisiae*, which serves as an eco-friendly example for producing compounds from traditional Chinese medical plants. Among the terpenes, monoterpenoids are usually bioactive compounds in plant essential oils. Gao et al. have reviewed the production of monoterpenoids using engineered yeasts. They especially described the application of protein engineering and structural biology strategies used to optimize key enzymes of the pathway. In the future, structural biology may enable highly efficient production of plant natural products using engineered yeasts (Cravens et al., 2019). Another application case is cocoa butter, which is the main component of chocolate. The application of yeasts for the biosynthesis of cocoa butter constituents has been achieved in the past few years, such as heterogeneously expression of cocoa lipid metabolic genes in *S. cerevisiae* (Wei et al., 2017a; Wei et al., 2017b; Wei et al., 2018). Wang et al. summarized recent development of producing cocoa butter equivalents using yeast, which might lead to the future production of yeast chocolate. RNA interference has been used for disease treatment and pest control, and yeast is one of the best hosts for the production of low-cost double-stranded RNA (dsRNA) for RNA interference applications (Zotti et al., 2018; Ahn et al., 2019). Guan et al. reviewed the advances in microbial dsRNA production systems including the use of yeast cells for their expression. Abbasi et al. updated recent advances in the production of sugar alcohols and functional sugars in *Yarrowia lipolytica*. Xylose is one of the most abundant sugars in nature and present in the side streams of lignocellulose processing (Wang et al., 2021). Zha et al. described the advances in the use of xylose as the substrate to produce natural products. Moreover, future challenges for the commercial production of natural products from xylose using engineered yeasts were discussed.

Tools and strategies to engineer yeasts for plant natural products are still in development. This research topic not only covers the synthetic biology technologies used for the production of natural products in yeast but also details several examples of valuable plant natural products produced in this host. This compendium of articles provides valuable insights for future developments. From our point of view, the integration of omics technologies, metabolic engineering, and synthetic biology strategies will accelerate the commercial production of bioactive plant compounds in the yeasts (Figure 1). In summary, this research topic highlights both the state of the art and the future perspectives of the biotechnological production of plant-derived natural products in yeast.

**FIGURE 1:**
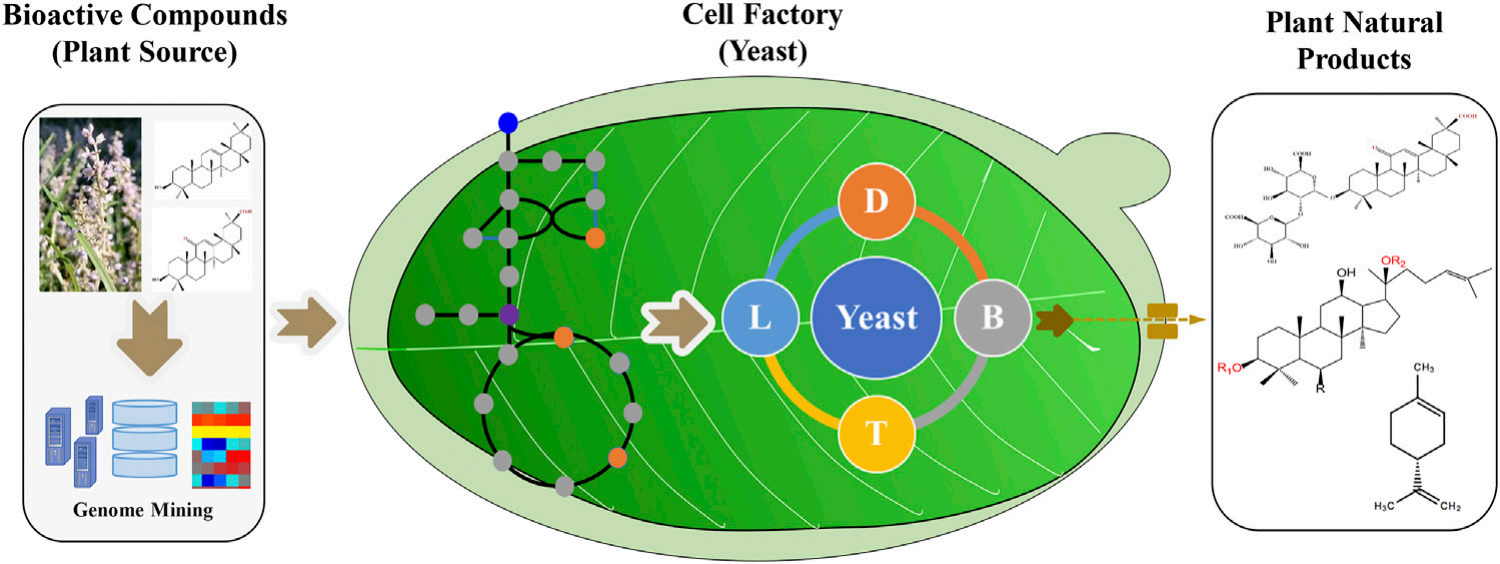
Yeast-based production of valuable plant natural products by metabolic engineering and synthetic biology strategies. The genes can be screened from plant omics data, and the synthetic biology tools help to engineer yeasts with the recovered enzymes and metabolism. The scale up strategies will further increase the titer, yield and rate of plant natural products.

## References

[B1] Ahn S. J. Donahue K. Koh Y. Martin R. R. Choi M. Y. (2019). Microbial-Based Double-Stranded RNA Production to Develop Cost-Effective RNA Interference Application for Insect Pest Management. Int. J. Insect Sci. 11, 1179543319840323. 10.1177/1179543319840323 31040730 PMC6482651

[B2] Cravens A. Payne J. Smolke C. D. (2019). Synthetic Biology Strategies for Microbial Biosynthesis of Plant Natural Products. Nat. Commun. 10, 2142. 10.1038/s41467-019-09848-w 31086174 PMC6513858

[B3] Ji Q. Mai J. Ding Y. Wei Y. Ledesma-Amaro R. Ji X.-J. (2020). Improving the Homologous Recombination Efficiency of *Yarrowia Lipolytica* by Grafting Heterologous Component from Saccharomyces Cerevisiae. Metab. Eng. Commun. 11, e00152. 10.1016/j.mec.2020.e00152 33294367 PMC7691175

[B4] Ma Y. Li J. Huang S. Stephanopoulos G. (2021a). Targeting Pathway Expression to Subcellular Organelles Improves Astaxanthin Synthesis in Yarrowia Lipolytica. Metab. Eng. 68, 152–161. 10.1016/j.ymben.2021.10.004 34634493

[B5] Ma Y. Li W. Mai J. Wang J. Wei Y. Ledesma-Amaro R. (2021b). Engineering Yarrowia Lipolytica for Sustainable Production of the Chamomile Sesquiterpene (−)-α-Bisabolol. Green. Chem. 23, 780–787. 10.1039/d0gc03180a

[B6] Nielsen J. Keasling J. D. (2016). Engineering Cellular Metabolism. Cell. 164, 1185–1197. 10.1016/j.cell.2016.02.004 26967285

[B7] Paddon C. J. Westfall P. J. Pitera D. J. Benjamin K. Fisher K. Mcphee D. (2013). High-Level Semi-Synthetic Production of the Potent Antimalarial Artemisinin. Nature. 496, 528–532. 10.1038/nature12051 23575629

[B8] Pickar-Oliver A. Gersbach C. A. (2019). The Next Generation of CRISPR-Cas Technologies and Applications. Nat. Rev. Mol. Cel Biol. 20, 490–507. 10.1038/s41580-019-0131-5 PMC7079207 31147612

[B9] Wang J. Liang J. Li Y. Tian L. Wei Y. (2021). Characterization of Efficient Xylanases From Industrial-Scale Pulp and Paper Wastewater Treatment Microbiota. AMB Expr. 11, 19. 10.1186/s13568-020-01178-1 PMC7815853 33464408

[B10] Wang P. Wei Y. Fan Y. Liu Q. Wei W. Yang C. (2015). Production of Bioactive Ginsenosides Rh2 and Rg3 by Metabolically Engineered Yeasts. Metab. Eng. 29, 97–105. 10.1016/j.ymben.2015.03.003 25769286

[B11] Wei Y. Bergenholm D. Gossing M. Siewers V. Nielsen J. (2018). Expression of Cocoa Genes in *Saccharomyces cerevisiae* Improves Cocoa Butter Production. Microb. Cell Fact. 17, 11. 10.1186/s12934-018-0866-2 29370801 PMC5784687

[B12] Wei Y. Gossing M. Bergenholm D. Siewers V. Nielsen J. (2017a). Increasing Cocoa Butter-Like Lipid Production of *Saccharomyces cerevisiae* by Expression of Selected cocoa Genes. AMB Expr. 7, 34. 10.1186/s13568-017-0333-1 PMC5293708 28168573

[B13] Wei Y. Siewers V. Nielsen J. (2017b). Cocoa Butter-Like Lipid Production Ability of Non-Oleaginous and Oleaginous Yeasts Under Nitrogen-Limited Culture Conditions. Appl. Microbiol. Biotechnol. 101, 3577–3585. 10.1007/s00253-017-8126-7 28168314 PMC5395598

[B14] Yang C. Li C. Wei W. Wei Y. Liu Q. Zhao G. (2020). The Unprecedented Diversity of UGT94-Family UDP-Glycosyltransferases in Panax Plants and Their Contribution to Ginsenoside Biosynthesis. Sci. Rep. 10, 15394. 10.1038/s41598-020-72278-y 32958789 PMC7506552

[B15] Zotti M. Dos Santos E. A. Cagliari D. Christiaens O. Taning C. N. T. Smagghe G. (2018). RNA Interference Technology in Crop protection against Arthropod Pests, Pathogens and Nematodes. Pest Manag. Sci. 74, 1239–1250. 10.1002/ps.4813 29194942

